# Supplementary material for: Fast DNA-PAINT imaging using a deep neural network
Source: Nat Commun. 2022 Aug 27;13:5047. doi: 10.1038/s41467-022-32626-0 (PMC9420107; doi:10.1038/s41467-022-32626-0)
Supplement: Supplementary file 1 — Supplementary Information [file 41467_2022_32626_MOESM1_ESM.pdf]

Fast DNA-PAINT imaging using a deep neural network

Kaarjel K. Narayanasamy[1,2], Johanna V. Rahm[2], Siddharth Tourani[3], Mike Heilemann[1,2*]
[1] Department of Functional Neuroanatomy, Institute for Anatomy and Cell Biology, Heidelberg University, Heidelberg, Germany
[2] Institute of Physical and Theoretical Chemistry, Goethe University Frankfurt, Frankfurt, Germany
[3] Computer Vision and Learning Lab, Heidelberg University, Heidelberg, Germany

Supplementary Information

Supplementary Note 1

Image similarity metrics

The HAWK Method for the Assessment of Nanoscopy (HAWKMAN) is an analysis algorithm to assess image similarity between two super-resolution images. HAWKMAN outputs a structure map, sharpening map, and an error map. This method suppresses nonlinearity in reconstructions between two images (reference and test) which arise due to intensity differences and degree of sampling, instead focusing on structural similarity. The structure map is an overlay of blurred, binarised, and skeletonised reference and test images which provides a final image with fine structural information. The sharpening map blurs and binarises the images to produce a final overlay which provides information on the local density of the structures. Density differences between the two images are described as artificial sharpening. Overlap between structures are presented in yellow, while a cyan region indicates an area that is not reconstructed in the test image, and magenta indicates artificial sharpening (false structures/loss of fine structures by the merging of features/increased density of structures) in the test image. The confidence map reports the confidence of a local region according to the cross-correlation of the structure and sharpening results, and intensity information. Both structure and sharpening maps also output a Pearson's Correlation Coefficient value [1].

Super-resolution quantitative imaging rating and reporting of error locations (SQUIRREL) is an analysis method that is based on the premise of comparing a super-resolution image (test image) to its diffraction-limited counterpart (reference image) to determine the completeness of the super-resolution reconstruction and intensity differences. The super-resolution image is resolution-scaled to match the resolution of the diffraction-limited image and a pixel-by-pixel absolute difference calculation between the diffraction-limited and resolution-scaled super-resolution image is performed to output an error map. In addition, the resolution-scaled Pearson coefficient (RSP) which is the Pearson's Correlation Coefficient and the resolution-scaled error (RSE) which is the root-mean-squared error (lower value indicates lower error) between both images are calculated [2]. We also performed a modified SQUIRREL analysis

here which uses the DeepSTORM predicted image as the reference image since it reports a higher decorrelation resolution value. By using this adapted approach, we were able to compare the images on a super-resolution scale (Supplementary Fig. 4).

Multi-Scale Structural Similarity Index (MS-SSIM) is a combined measure of the luminance, contrast, and structure similarity between two images calculated at different image scales. [3].

Supplementary Note 2

The minimum number of frames required to obtain a completely reconstructed image using DeepSTORM was determined by comparing predicted images generated from 50 to 2,000 frame lengths to the GT image and obtaining structural similarity values using HAWKMAN (Supplementary Fig. 1). We found that at a concentration of 5 or 10 nM, a completely reconstructed image could be obtained with only 400 frames (dotted lines) for both α-tubulin and TOM20. The performance of the single-molecule localisation algorithm Picasso was also tested using a 10 nM TOM20 high-density dataset (Supplementary Fig. 2) which showed incomplete reconstruction in TOM20 regions (white circles).

An α-tubulin-labelled region in tissue with 1D (left) and 2D (right) structures from the same image are compared side by side (Supplementary Fig. 3a). The structure and sharpening map both show that 1D α-tubulin filaments are predicted well by DeepSTORM (yellow structures; yellow arrows) but the strong presence of local cyan structures (white arrows) indicates 2D α-tubulin bundles are lacking structural density in the predicted images (Supplementary Fig. 3bc). The confidence maps corroborate these findings where low reconstruction correlation in the 2D regions and good correlation in the 1D regions are evident (Supplementary Fig. 3d).

Image prediction similarity was assessed for α-tubulin- and TOM20-labelled images using HAWKMAN, SQUIRREL, MS-SSIM, and decorrelation resolution (Supplementary Fig. 4). The overlay between GT and predicted images (5, 10, or 20 nM) indicate either structural agreement (white), denser GT structures (cyan) or denser predicted structures (magenta) (Supplementary Fig. 4a). Visual comparisons indicate that 5 nM and 10 nM predictions of α-tubulin are highly similar (Supplementary Fig. 4a i-ii) whereas a noticeable difference is observed in 20 nM where the cyan in structurally dense regions are more prominent (Supplementary Fig. 4a iii; yellow arrows), suggesting incomplete reconstruction of the predicted image. Here, DeepSTORM loses its prediction quality at 20 nM for dense 2D α-tubulin structures while maintaining the reconstruction of 1D structures. The comparison of a particularly dense 2D structural region of an axon [4] was chosen to observe the challenges of our NN model when applied to a high-density hotspot. GT (cyan) and predicted 5, 10 and 20 nM concentration (magenta) images were assessed (Supplementary Fig. 4b). We found the prediction quality for extremely dense 2D structures was low with pixelated rendering artefacts, exacerbated by increasing imager strand concentrations (Supplementary Fig. 4b i-iv). SQUIRREL error maps indicate larger errors with increasing concentration of imager strands and dissimilarities in structures can be seen in blue and green regions, while yellow regions indicate differences in intensity (Supplementary Fig. 4b v-vii).

Visual inspection of TOM20 predicted images compared to GT indicate that an imager strand concentration of 5 nM is not sufficient to completely reproduce mitochondrial structures (high cyan density), whereas at 10 nM there is better similarity between GT and the predicted image (Supplementary Fig. 4c i-ii). At 20 nM hallucination artefacts are being predicted in the DeepSTORM image which are not found in GT, seen as an increase in magenta structures (Supplementary Fig. 4c iii). A magnified region of a single mitochondria shows that the GT image is finer and more punctate compared to the larger and diffuse points of the predicted images (Supplementary Fig. 4d i-iv). While the mitochondrial structure and shape were effectively reconstructed in all three predicted imager strand concentrations, the 5 nM imager strand prediction is incomplete (yellow arrows, Supplementary Fig. 4d ii). At 10 nM, the mitochondrial shape is more defined and better reproduced, and at 20 nM hallucination artefacts (features that do not exist in GT) are formed (magenta arrows, Supplementary Fig. 4d iv). The error maps show very subtly that 10 nM imager strand concentration has the lowest structural error. Strong yellow regions dotted around the structure reflect differences in intensity rather than structural inconsistencies possibly due to differences in emitter photon intensity or degree of sampling between the datasets during image acquisition (Supplementary Fig. 4d v-vii; white arrows; [1].
The quality of DeepSTORM predicted structures compared to GT were quantitatively assessed (Supplementary Fig. 4e). For SQUIRREL analysis, α-tubulin showed slightly higher RSP values for 5 nM imager strand concentrations while no difference was observed for TOM20 RSP values in all imager strand concentrations. α-tubulin and TOM20 both have the lowest RSE at 5 nM (*p* α-tubulin = 0.02; ANOVA). This suggests that an increase in imager strand concentration contributes to higher background fluorescence which affects image prediction quality. The MS-SSIM for α-tubulin had the highest structural similarity at 5 nM imager strand whereas in TOM20-labelled structures only 20 nM was unsuitable for prediction. In the HAWKMAN analysis for both structural reconstruction and artificial sharpening, α-tubulin at 5 nM performed well (p = 0.02; ANOVA) whereas TOM20 had comparable structural correlation for 5 and 10 nM and better sharpening correlation at 10 nM. Decorrelation resolution for both α-tubulin and TOM20 are lower in all predicted images compared to their respective GT images (~35 nm) by approximately 10 nm.

We applied SQUIRREL analysis on the super-resolved low-density emitter (0.5 nM, 10,000 frames, DNA-PAINT) and high-density predicted DeepSTORM images (5, 10, 20 nM; 400 frames) against their respective diffraction-limited DNA-PAINT frames obtained by z-projection (Supplementary Fig. 5). The low-density emitter image showed the best RSP value and the lowest RSE compared to DeepSTORM predicted images, also reflected in the error map showing high image correlation (Supplementary Fig. 5a iii). With the increase in imager strand concentrations for predicted images, the RSP, RSE, and error map become worse. While the filamentous 1D structures on the left side of the images are largely unchanged in the error map, the prediction quality of 2D dense structures (right side) become noticeably poor (Supplementary Fig. 5a iv-vi). Similar to α-tubulin, the low-density emitter image for TOM20 showed the best outcome with the highest RSP and lowest RSE value compared to predicted images (Supplementary Fig. 5b iii-vi). Again, both the RSP and RSE values suffered with increasing imager strand concentrations, although the difference between 5 and 10 nM was low

in mitochondrial structures. Image prediction quality suffers with increasing imager strand concentrations and may be attributed to excessive overlap of emitters and high background fluorescence. The prediction is also affected by structure dimensionality, whereby 1D structures were predicted better than 2D structures.

We sought to determine the generality of our model by extending the range of protein target prediction using 2-target Exchange-PAINT. We found that our model could predict nanostructures on a scale of ~100 nm for Bassoon and Homer structures (Supplementary Fig. 7ab) and differentiate between cells in MNTB tissue such as neurons and astrocytes (Supplementary Fig. 7cd). Furthermore, the model was stable over many months since training provided the optical setup was unchanged (Supplementary Fig. 8).

A post-processing extension of DeepSTORM functionality was developed in the ZeroCostDL4Mic platform which extracts localisations from points in the predicted DeepSTORM image. Benefits of having SMLM localisations are the ability to perform drift correction, rendering a super-resolution image with different algorithms, and performing coordinate-based image analysis. We briefly studied the localisation output of the post-processing function in the Colab notebook. An experimental and artificial high-density TOM20 dataset was used for image prediction in DeepSTORM and subsequently post-processed to extract DeepSTORM localisations. The localisations were rendered in Picasso using the same rendering method used for the GT. The image similarity of GT, DeepSTORM predicted image, and DeepSTORM localisations rendered in Picasso were compared using HAWKMAN and MS-SSIM (Supplementary Fig. 10). In general, there was a very slight increase by 0.02 in the similarity metrics of GT vs DeepSTORM localisations in the experimental dataset (Supplementary Fig. 10a). Although this difference is negligible, generally an improvement in the image analysis metrics can be attributed to the similar rendering method in Picasso using the One-Pixel Blur.

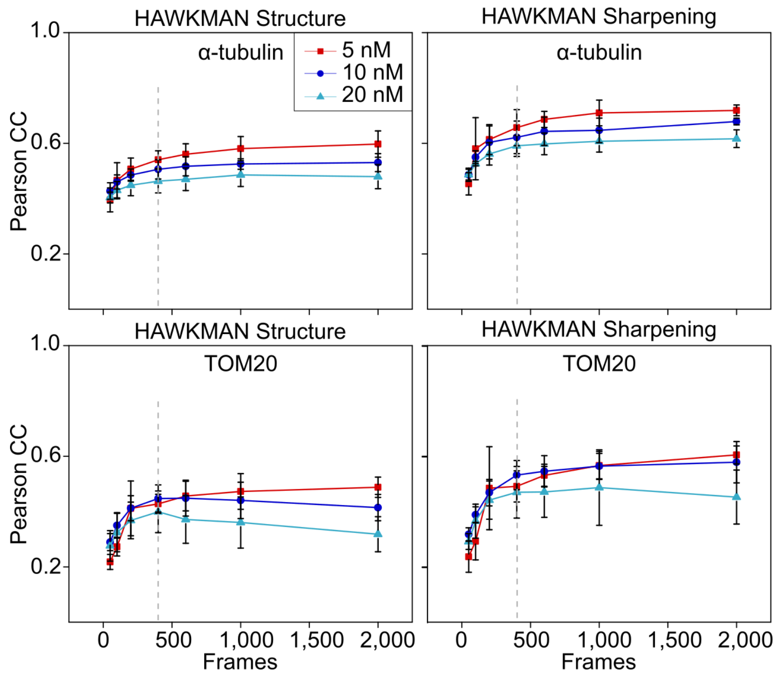

Supplementary Figure 1: Optimization of frame length for complete image reconstruction using DeepSTORM. High density frames at 5 (red), 10 (blue), and 20 (cyan) nM imager strand concentrations at frame lengths of 50, 100, 200, 400, 600, 1,000, and 2,000 were predicted with DeepSTORM and the predicted image similarity evaluated against GT images. HAWKMAN image similarity metric was applied to the whole image to determine the minimum number of frames required for complete image reconstruction. Vertical stippled line marks frame length at 400; n = 3 images per data point, error bars = SD. Source data are provided as a Source Data file.

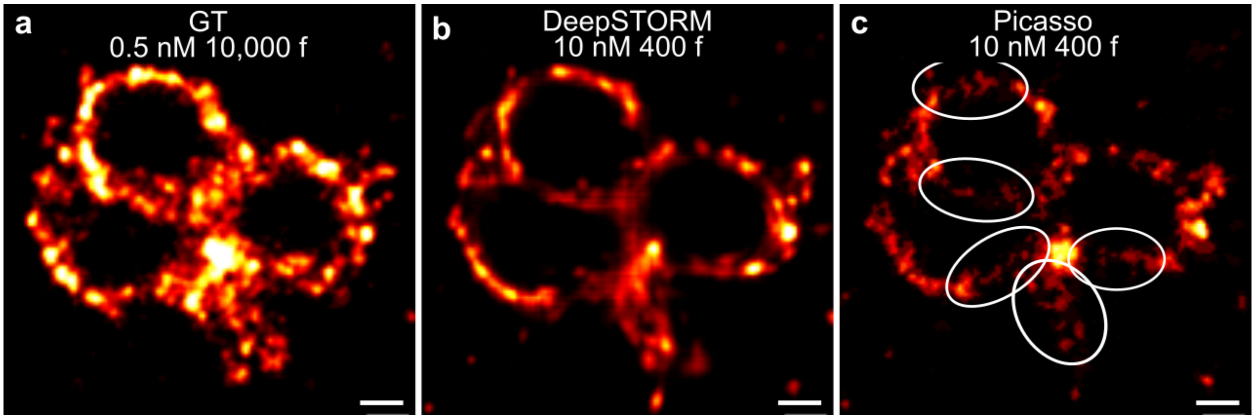

Supplementary Figure 2: Predicting a high-density dataset with single-emitter localisation algorithm Picasso. A magnified TOM20 (**a**) ground truth (GT) low-density image (0.5 nM 10,000 frames) rendered with Picasso, (**b**) DeepSTORM predicted high-density image (10 nM 400 frames), and (**c**) Picasso-rendered high-density image (10 nM 400 frames). Circled regions in c highlight areas of incomplete structural reconstruction. N = 1 cropped ROI; scale bars 0.2 μm.

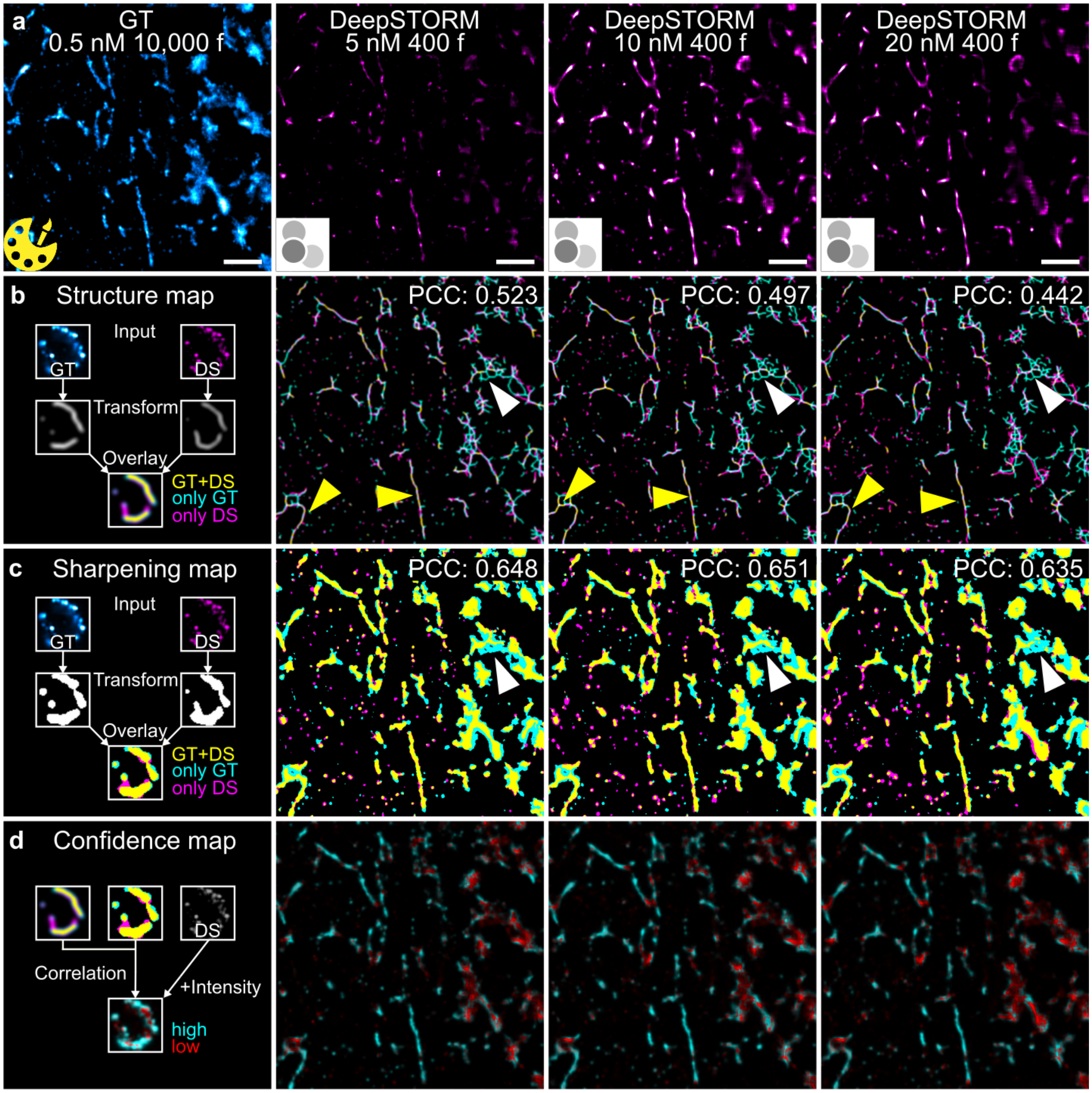

Supplementary Figure 3: Quantitative analysis of image similarity between ground truth (GT) and predicted super-resolution images using HAWKMAN. (**a**) GT (cyan; yellow Picasso icon) and DeepSTORM predicted images (magenta; DeepSTORM icon) of an α-tubulin-labelled structure recorded for imager strand concentrations of 0.5, 5, 10, and 20 nM. (**b**) Structure map with Pearson correlation coefficient (PCC) indicating regions of good overlap between GT vs predicted image (yellow structures; yellow arrows), denser GT structures (cyan structures; white arrows) or denser DeepSTORM predicted structures (magenta structures). (**c**) Sharpening map indicating regions of artificial sharpening with the same colour scheme as the structure map. (**d**) Confidence map highlighting structures of high confidence (cyan) and low confidence (red). (**b**, **c**, & **d**; first column) Schematic explaining HAWKMAN maps. HAWKMAN applied to n = 1 ROI (**a-d**); scale bars 1 μm.

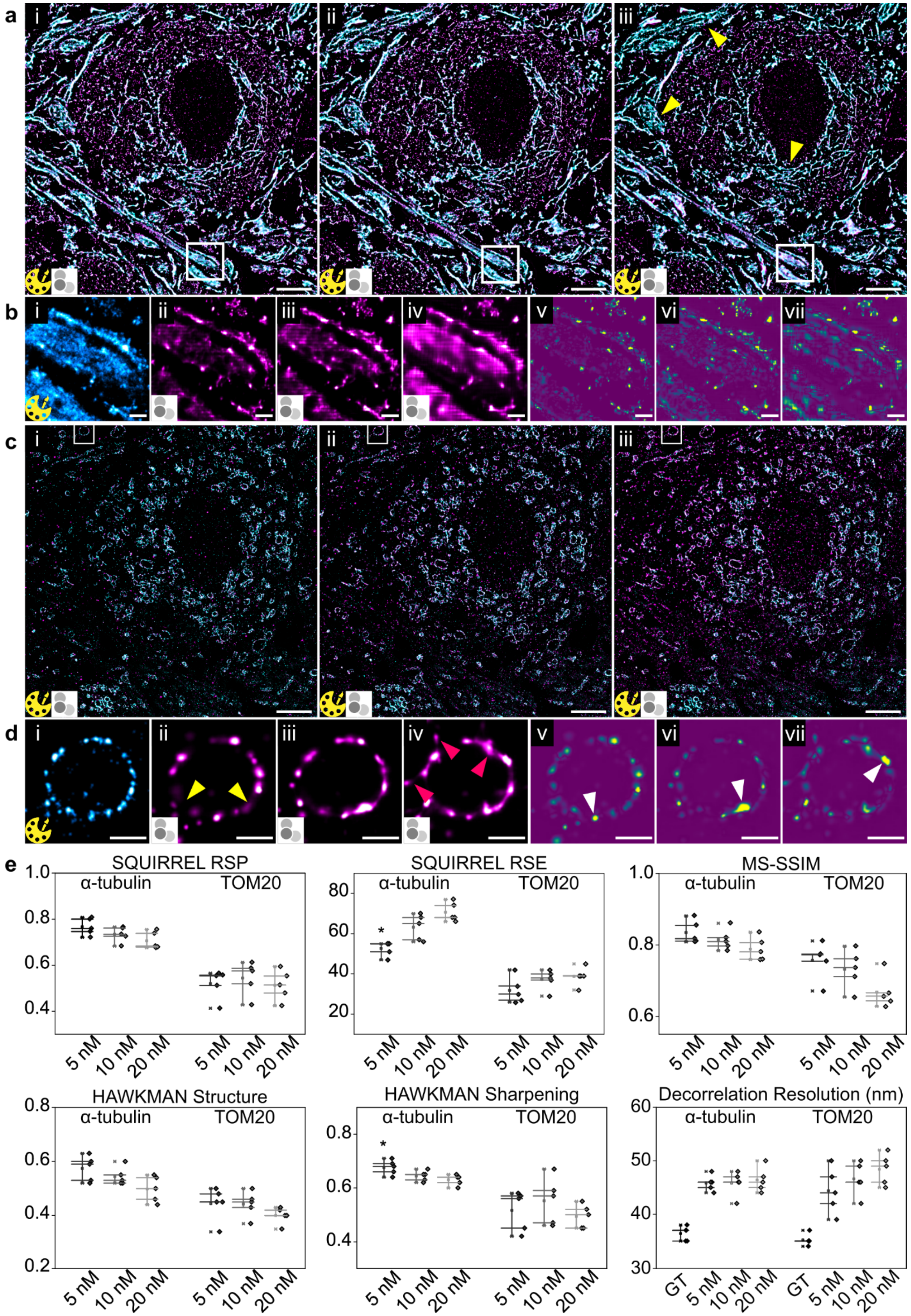

a
i
ii
iii
b
i
ii
iii
iv
v
vi
vii
c
i
ii
iii
d
i
ii
iii
iv
v
vi
vii
e
SQUIRREL RSP
α-tubulin
TOM20
5 nM
10 nM
20 nM
SQUIRREL RSE
MS-SSIM
HAWKMAN Structure
HAWKMAN Sharpening
Decorrelation Resolution (nm)
GT

Supplementary Figure 4: Quantitative assessment tools for the analysis of predicted super-resolution images. (**a**) Overlay of GT α-tubulin-labelled images (0.5 nM, 10,000 frames; cyan; yellow Picasso icon) with (**i**) 5 nM, (**ii**) 10 nM, and (**iii**) 20 nM DeepSTORM predicted images (400 frames; magenta; DeepSTORM icon). (**b**) A magnified region of a very dense axon structure from a with (**i**) GT; cyan, (**ii**) 5 nM, (**iii**) 10 nM, and (**iv**) 20 nM predicted images (magenta). SQUIRREL error map between GT and (**v**) 5 nM, (**vi**) 10 nM, and (**vii**) 20 nM predicted images. (**c**) Same as **a** for TOM20. (**d**) Same as b for TOM20. (**e**) Boxplot (median as centre line, mean as centre point, top and bottom lines as Q1 and Q3, whiskers are outliers) with random jittered data points of DeepSTORM predicted image quality assessment against GT image using RSP (Resolution Scaled Pearson) and RSE (Resolution Scaled Root Mean Squared Error) from SQUIRREL; MS-SSIM (Multi-Scale Structural Similarity Index); Pearson Correlation-based (PCC) structure similarity and image sharpening with HAWKMAN; and decorrelation resolution; n = 5 images per condition. Yellow arrows pointing to cyan regions indicate incompletely predicted structures. White arrows indicate structures with high intensity differences. Magenta arrows indicate hallucination artefacts. Asterisks indicate statistical significance at <0.05 using One-way ANOVA. Scale bar 4 µm (**a** & **c**), 0.5 µm (**b** & **d**). Source data are provided as a Source Data file.

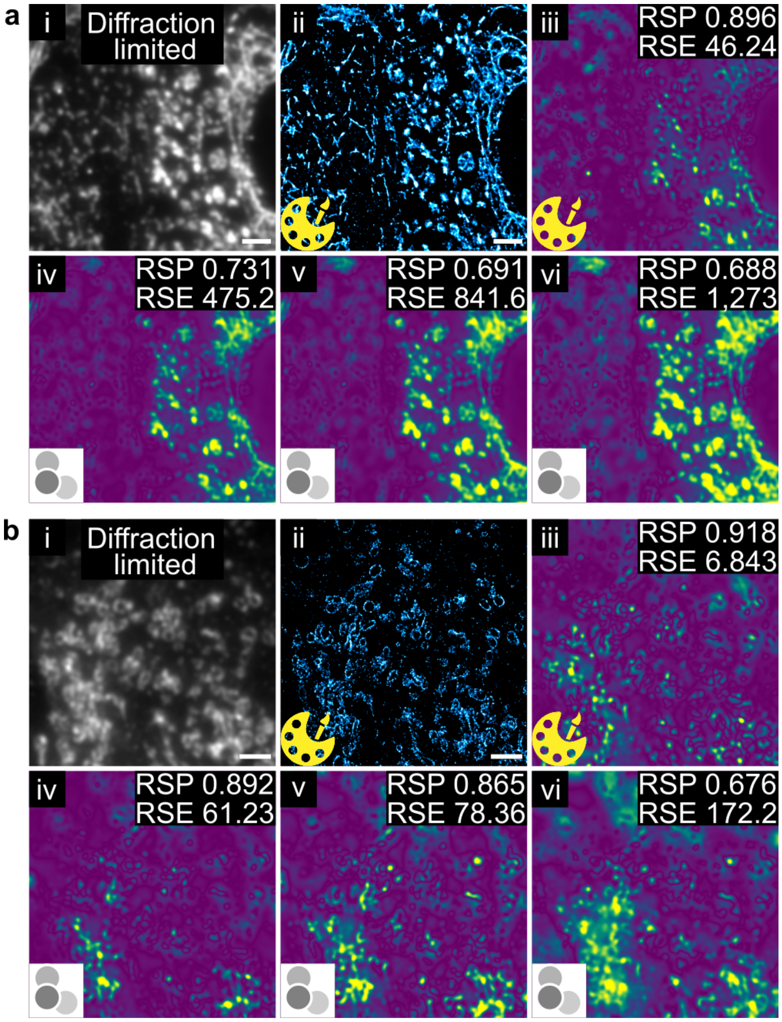

Supplementary Figure 5: SQUIRREL analysis between super-resolution and diffraction-limited images to measure nanostructure reconstruction. (**a**) α-tubulin-labelled structure with (**i**) a diffraction-limited image (grey) and (**ii**) corresponding DNA-PAINT low-density emitter super-resolution image (0.5 nM imager strands for 10,000 frames; cyan; Yellow Picasso icon). SQUIRREL analysis (**iii**) comparing **ii** and **i**, and comparing DeepSTORM predicted images (DeepSTORM icon) with diffraction-limited images (400 frames) for (**iv**) 5 nM (**v**) 10 nM and (**vi**) 20 nM imager strands (**b**) Same as **a** for TOM20-labelled structures. SQUIRREL applied to ROI of n = 1 (**a** & **b**); scale bar 2 μm.

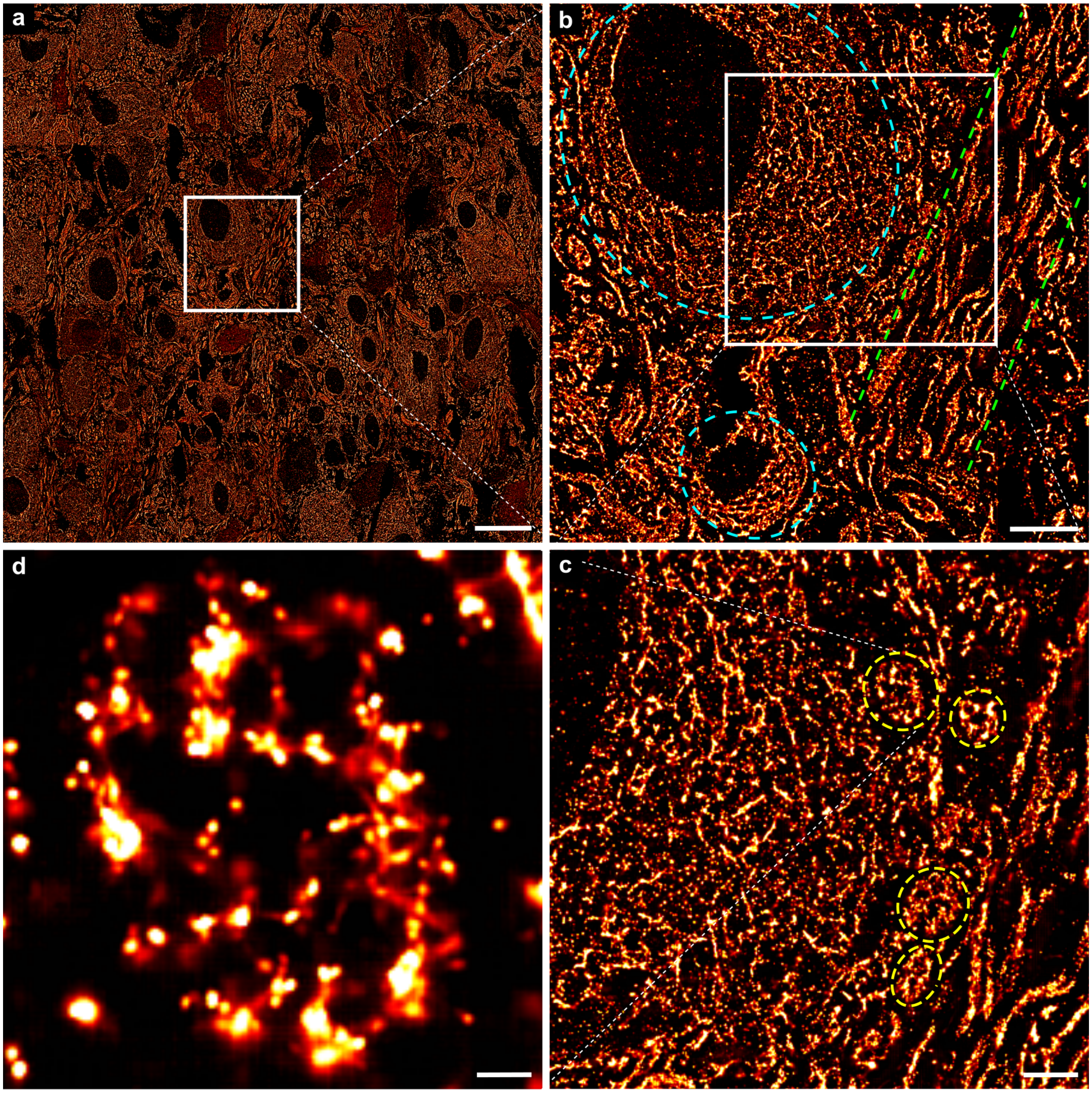

Supplementary Figure 6: Magnified super-resolution large-sample image with DeepSTORM DNA PAINT to observe nanostructures. (**a**) A large-ROI super-resolution α-tubulin image (in Fig. 5). (**b**) Magnification of a region in **a** showing two cells (cyan stippled lines) and a column of axon filaments (green stippled lines). (**c**) Magnification of part of the cell and cross-sections of axon bundles (yellow stippled lines). (**d**) Cross-section of a single axon bundle showing the nanostructure features that can be extracted by large sample super-resolution imaging. N = 1 tissue sample; scale bar (**a**) 20 μm, (**b**) 5 μm, (**c**) 2 μm, (**d**) 0.25 μm.

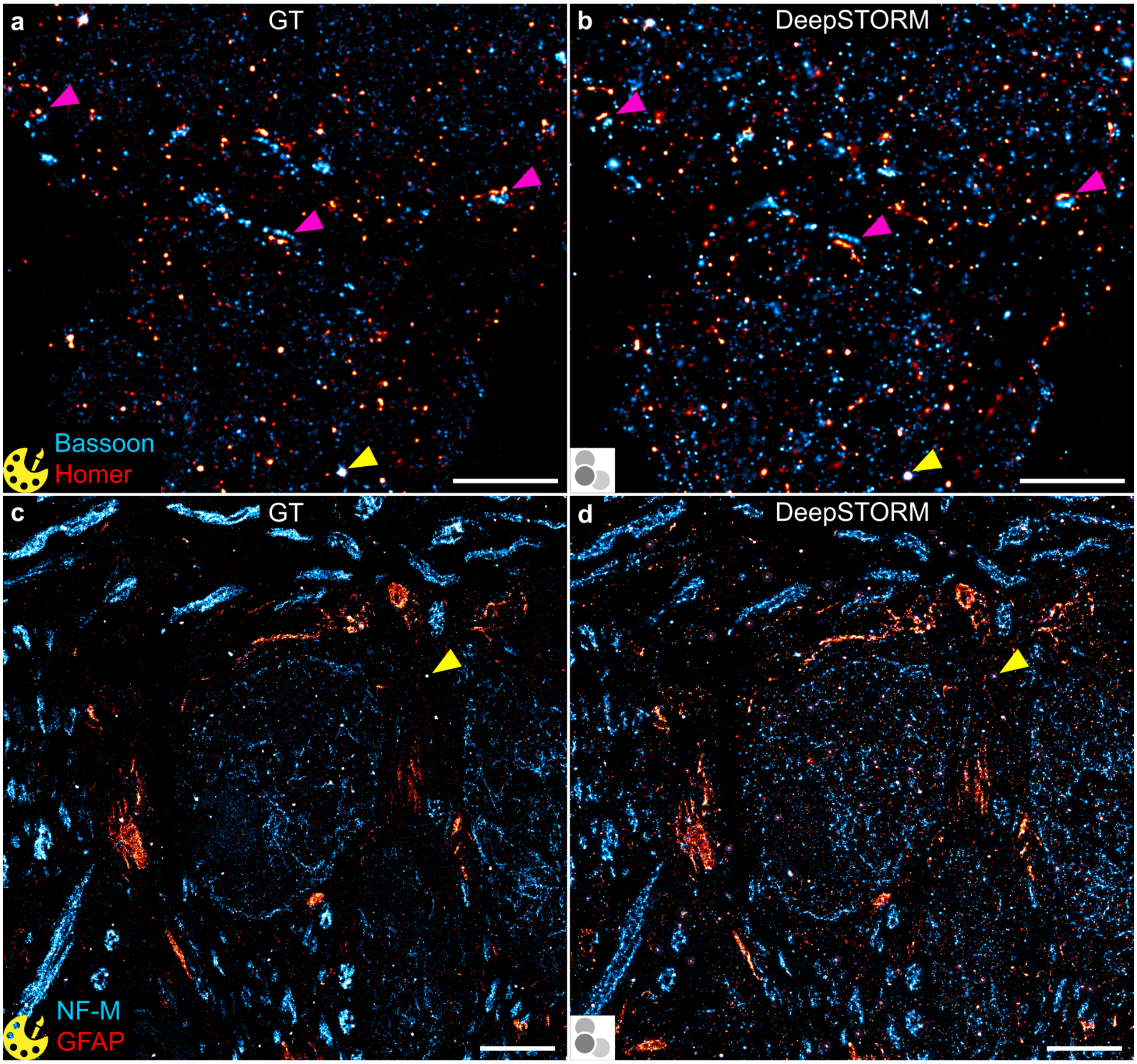

Supplementary Figure 7: One DeepSTORM model predicts different protein structures. Different protein targets were imaged using 2-target Exchange-PAINT to observe DeepSTORM image prediction using the same model on different structures. (**a**) Ground truth (GT) and (**b**) predicted image of Bassoon (cyan; 5 nM P1) and Homer (red; 5 nM P5). Magenta arrows indicate typical Bassoon and Homer juxtaposed nanostructures in the active zone of synapses. (**c**) GT and (**d**) predicted image of neurofilament M (NF-M; cyan; 5 nM P1) and glial fibrillary acidic protein (GFAP; red; 1 nM R1) labelling neurons and astrocytes, respectively. Fiducial markers are used to register multi-target images (yellow arrows). N = 1 image (**a-d**); scale bar (a & b) 2 µm, (c & d) 5 µm.

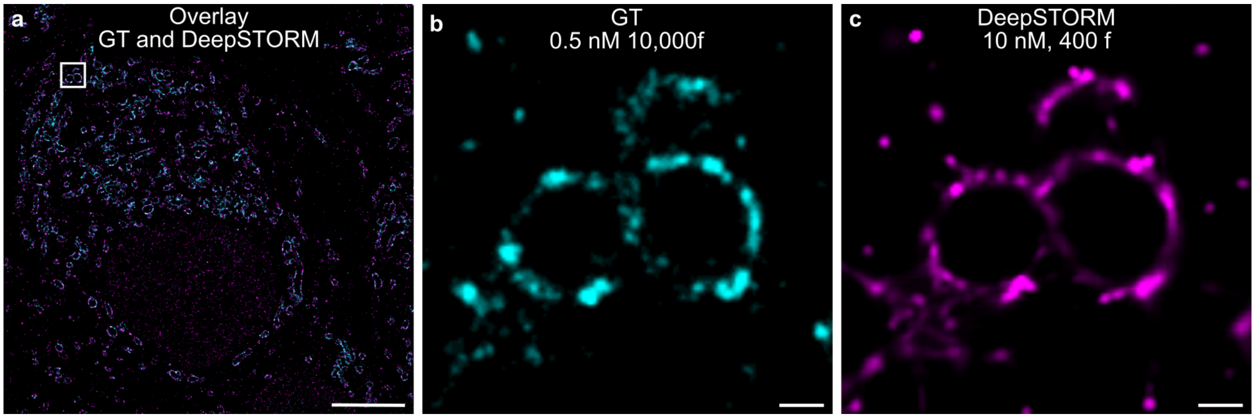

Analysis Metrics		5 nM	10 nM	20 nM
HAWKMAN	Structure<br>Sharpening	0.51<br>0.55	0.47<br>0.56	0.44<br>0.49
SQUIRREL	RSP<br>RSE	0.55<br>59	0.54<br>62	0.51<br>61
MS-SSIM	-	0.71	0.67	0.65
Decorrelation resolution	38 nm (GT)	42 nm	42 nm	43 nm

Supplementary Figure 8: Longevity of the trained model. A TOM20 dataset was imaged and predicted with DeepSTORM months after training the model. (**a**) Overlay of ground truth (GT; 0.5 nM, 10,000 frames; cyan) with DeepSTORM predicted image (10 nM, 400 frames; magenta). A magnified region of (**b**) GT and (**c**) predicted image. (Table) Image similarity metrics on the whole image for 5, 10 and 20 nM imager strand concentrations. N = 1 image; scale bars (**a**) 5 μm, (**b** & **c**) 0.2 μm.

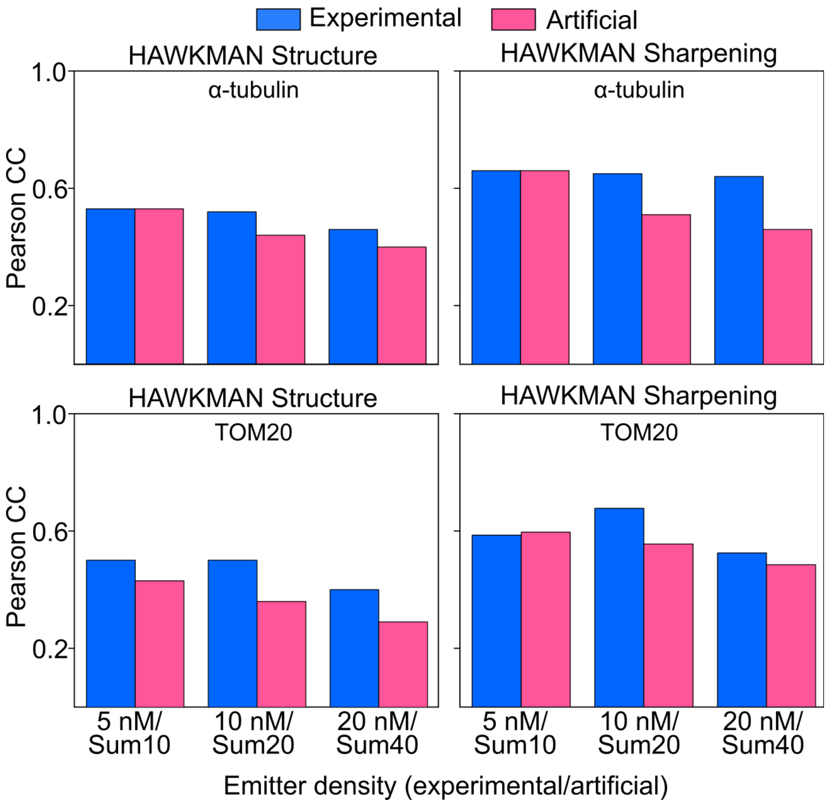

Supplementary Figure 9: Comparison of experimental high-density data to ground truth (GT) data, and of artificially-generated high-density data to GT data. DeepSTORM predicted images from experimental datasets (5, 10, and 20 nM imager strands; 400 frames; blue) and artificially-generated datasets generated from imaging data recorded with 0.5 nM imager strand concentration (Sum10, Sum20, and Sum40; 400 frames; pink) for α-tubulin and TOM20 were compared to GT images (0.5 nM, 10,000 frames) using HAWKMAN. Image similarity metrics on the whole image, n = 1. Source data are provided as a Source Data file.

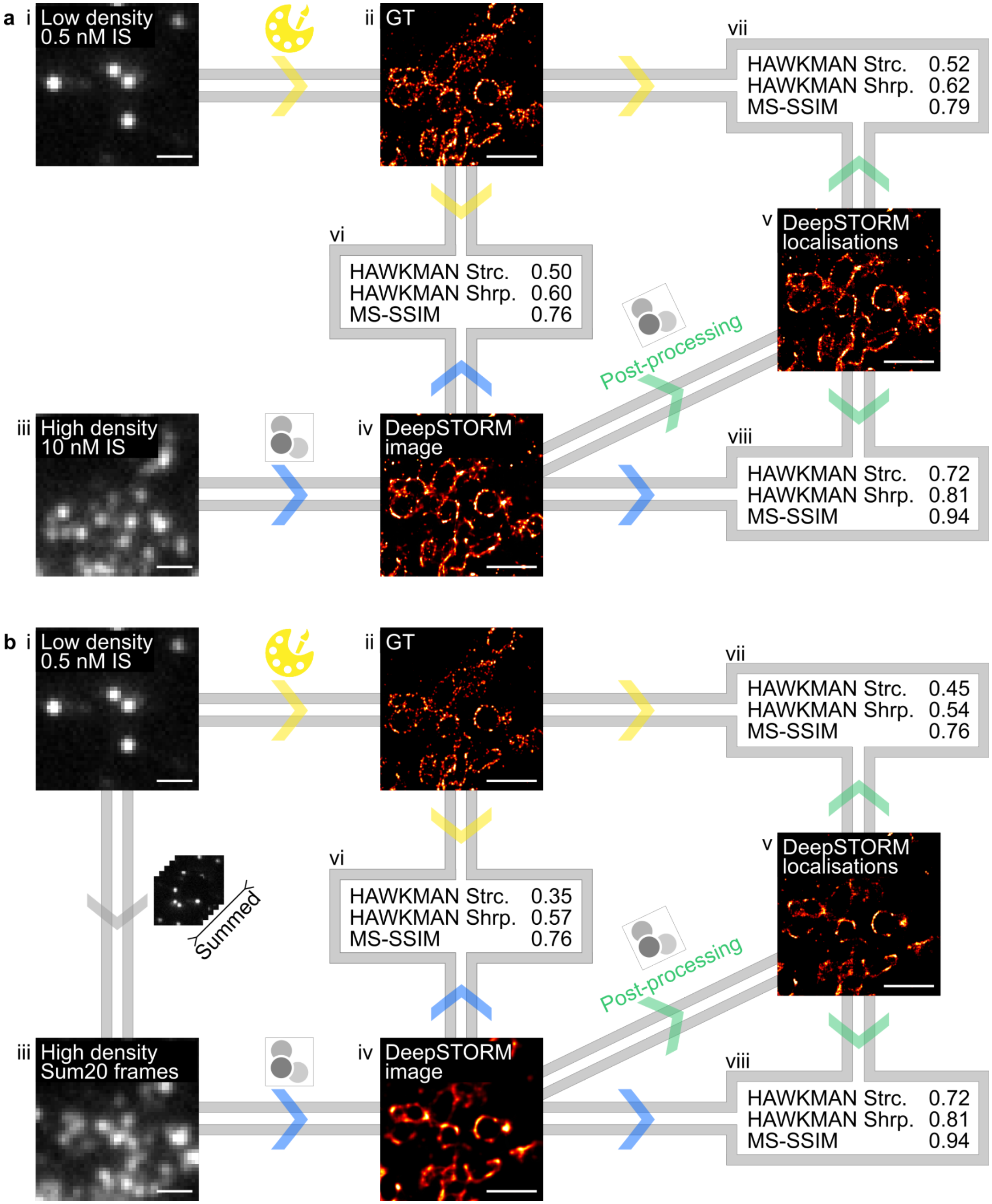

Supplementary Figure 10: DeepSTORM extracts single-molecule localizations for super-resolution image reconstruction. (**a**) From a TOM20-labelled sample, (**i**) low emitter density frames were imaged (0.5 nM 10,000 frames) and (**ii**) a GT image was reconstructed with Picasso (Yellow Picasso icon; yellow arrows). (**iii**) High density frames were obtained by image acquisition of the sample with 10 nM imager strands. (**iv**) The high density dataset was used to

predict an image with the DeepSTORM model (blue arrows) and post-processing was performed in DeepSTORM on the predicted image to extract emitter localisations (green arrows). (**v**) DeepSTORM-extracted localisations were rendered in Picasso. Image similarity was analysed using HAWKMAN and MS-SSIM for (**vi**) GT vs DeepSTORM predicted image, (**vii**) GT vs DeepSTORM-extracted localisations, and (**viii**) DeepSTORM predicted image vs DeepSTORM-extracted localisations. (**b**) Similar to (**a**) except (**iii**) artificially-generated high density frames were prepared by summing up groups of 20 frames from the low density dataset in **i**. Yellow arrows indicate GT image processing, blue arrows indicate DeepSTORM image processing, and green arrows indicate DeepSTORM localisation processing. Image similarity metrics performed on the whole image, n = 1; scale bar 1 µm.

Supplementary Table 1: Parameters for DeepSTORM training and prediction.

Training

Neural network	Raw frame size	Emitter density of raw frames	Patch size	# of patches	Emitter density
DeepSTORM v1.12	512 x 512	0.028 em/µm²	16 x 16	30,000	2 em/µm²

Server	GPU	# of epochs	Batch size	Learning rate	Upsampling factor	Percentage validation	Training time
Google Colab Pro	Tesla V100	100	256	$1e^{-5}$	8	0.15	35 min

Prediction

Neural network	Server	GPU	Frame size	# of frames	Prediction time
DeepSTORM v1.13	Google ColabPro/Colab	Tesla P100/ Tesla K80	512 x 512	400	7 - 25 min

References

1. Marsh, R. J. *et al.* Sub-diffraction error mapping for localisation microscopy images. *Nat. Commun.* **12**, 5611 (2021).
2. Culley, S. *et al.* Quantitative mapping and minimization of super-resolution optical imaging artifacts. *Nat. Methods* **15**, 263–266 (2018).
3. Wang, Z., Simoncelli, E. P. & Bovik, A. C. Multiscale structural similarity for image quality assessment. *The Thrity-Seventh Asilomar Conference on Signals, Systems & Computers,*

2003 (2003) doi:10.1109/acssc.2003.1292216.

4. Narayanasamy, K. K. *et al.* Visualizing Synaptic Multi-Protein Patterns of Neuronal Tissue With DNA-Assisted Single-Molecule Localization Microscopy. *Front. Synaptic Neurosci.* **13**, 671288 (2021).
