# Supplementary material for: Fast DNA-PAINT imaging using a deep neural network
Source: Nat Commun. 2022 Aug 27;13:5047. doi: 10.1038/s41467-022-32626-0 (PMC9420107; doi:10.1038/s41467-022-32626-0)
Supplement: Supplementary file 3 — Reporting Summary [file 41467_2022_32626_MOESM3_ESM.pdf]

nature portfolio

Corresponding author(s): Mike Heilemann

Last updated by author(s): Aug 5, 2022

Reporting Summary

Nature Portfolio wishes to improve the reproducibility of the work that we publish. This form provides structure for consistency and transparency in reporting. For further information on Nature Portfolio policies, see our Editorial Policies and the Editorial Policy Checklist.

Statistics

For all statistical analyses, confirm that the following items are present in the figure legend, table legend, main text, or Methods section.

n/a	Confirmed	
☐	☒	The exact sample size (*n*) for each experimental group/condition, given as a discrete number and unit of measurement
☐	☒	A statement on whether measurements were taken from distinct samples or whether the same sample was measured repeatedly
☒	☐	The statistical test(s) used AND whether they are one- or two-sided<br>*Only common tests should be described solely by name; describe more complex techniques in the Methods section.*
☒	☐	A description of all covariates tested
☒	☐	A description of any assumptions or corrections, such as tests of normality and adjustment for multiple comparisons
☐	☒	A full description of the statistical parameters including central tendency (e.g. means) or other basic estimates (e.g. regression coefficient) AND variation (e.g. standard deviation) or associated estimates of uncertainty (e.g. confidence intervals)
☒	☐	For null hypothesis testing, the test statistic (e.g. *F*, *t*, *r*) with confidence intervals, effect sizes, degrees of freedom and *P* value noted<br>*Give P values as exact values whenever suitable.*
☒	☐	For Bayesian analysis, information on the choice of priors and Markov chain Monte Carlo settings
☒	☐	For hierarchical and complex designs, identification of the appropriate level for tests and full reporting of outcomes
☐	☒	Estimates of effect sizes (e.g. Cohen's *d*, Pearson's *r*), indicating how they were calculated

Our web collection on statistics for biologists contains articles on many of the points above.

Software and code

Policy information about availability of computer code

Data collection	Single-molecule data was collected using µManager 2.0-gamma.
Data analysis	Raw imaging data was analyzed with the Picasso software (version 0.2.8), which is published and publicly available. For DeepSTORM analysis, the implementation into ZeroCostDL4Mic on a Google Colab notebook (version 1.12 and 1.13) was used; this tool and documentation are published, and freely available. A custom code for binning single-molecule data was developed, and made freely accessible on https://github.com/JohannaRahm/ImageSumming (version 220620). OriginPro 2019 (version 9.6.0.172) were used for graphing and statistical analyses. Python version 3.8.3 and Fiji version v1.53p.

For manuscripts utilizing custom algorithms or software that are central to the research but not yet described in published literature, software must be made available to editors and reviewers. We strongly encourage code deposition in a community repository (e.g. GitHub). See the Nature Portfolio guidelines for submitting code & software for further information.

Data

Policy information about availability of data

All manuscripts must include a data availability statement. This statement should provide the following information, where applicable:

- Accession codes, unique identifiers, or web links for publicly available datasets
- A description of any restrictions on data availability
- For clinical datasets or third party data, please ensure that the statement adheres to our policy

(1) The raw data used to create high-density patches for NN training, (2) summed high-density patches with localisation list to train the NN, (3) the trained model and weights, (4) high-density movies of α-tubulin and TOM20 from which high-resolution images were predicted with the neural network, (5) low-density movies of 0.5 nM imager strands concentration, (6) rendered ground truth images of α-tubulin and TOM20, (6) Bassoon and Homer ground truth images with corresponding

high-density frames, and (7) large stitched super-resolution image obtained with the NN have been deposited in the Zenodo database under accession code https://doi.org/10.5281/zenodo.6966132. Source data are provided with this paper.

Field-specific reporting

Please select the one below that is the best fit for your research. If you are not sure, read the appropriate sections before making your selection.

- [x] Life sciences
- [ ] Behavioural & social sciences
- [ ] Ecological, evolutionary & environmental sciences

For a reference copy of the document with all sections, see nature.com/documents/nr-reporting-summary-flat.pdf

Life sciences study design

All studies must disclose on these points even when the disclosure is negative.

Sample size	A sample size of n = 5 images for two different targets each (alpha-tubulin and TOM20) with morphologically distinct structures was used to determine the performance of the trained model using image similarity metrics (Supplementary Figure S4). Bassoon image-based cluster analysis of n = 75 clusters from 3 tissue samples was used for linear fitting to determine the performance of the trained model (Figure 4).
Data exclusions	No data was excluded from the analysis.
Replication	The manuscript compares single-molecule DNA-PAINT images with high-density DeepSTORM predictions. Next to visual inspection, we used various image comparison methods and identifiers. Repetitive experiments were undertaken to extract quantitative information and are reported in Supplementary Figure S4. Image-based cluster analysis is detailed in Methods and reported in Figure 4.
Randomization	All tissue samples were processed and imaged with the same conditions and analysed equally, therefore there was no requirement for performing randomisation.
Blinding	Blinding is not relevant to this study as all treatment data were directly compared to their respective control images.

Reporting for specific materials, systems and methods

We require information from authors about some types of materials, experimental systems and methods used in many studies. Here, indicate whether each material, system or method listed is relevant to your study. If you are not sure if a list item applies to your research, read the appropriate section before selecting a response.

Materials & experimental systems

n/a	Involved in the study	
[ ]	[x]	Antibodies
[x]	[ ]	Eukaryotic cell lines
[x]	[ ]	Palaeontology and archaeology
[ ]	[x]	Animals and other organisms
[x]	[ ]	Human research participants
[x]	[ ]	Clinical data
[x]	[ ]	Dual use research of concern

Methods

n/a	Involved in the study	
[x]	[ ]	ChIP-seq
[x]	[ ]	Flow cytometry
[x]	[ ]	MRI-based neuroimaging

Antibodies

Antibodies used	Tissue samples were labelled with primary antibodies against α-tubulin-mouse (T6199, Sigma-Aldrich; clone DM1A; dilution 1:500), TOM20-rabbit (sc-11415, Santa Cruz Biotechnology; dilution 1:80), Bassoon-mouse (SAP7F407, Enzo Life Sciences; clone SAP7F407; dilution 1:500), Homer1-rabbit (160003, Synaptic Systems; dilution 1:500), Glial Fibrillary Acidic Protein-chicken (GFAP; 173006; Synaptic Systems; dilution 1:500), or Neurofilament M-mouse (NF-M; 171241, Synaptic Systems; clone 103H5A1; dilution 1:500).<br>This information is also provided in the Methods section.
Validation	We used commercial antibodies and refer to tests provided by the vendors The validation by the vendors are listed below.<br>1. α-tubulin-mouse (T6199, Sigma-Aldrich)<br>(a) Vendor website: https://www.sigmaaldrich.com/DE/en/product/sigma/t6199<br>(b) Quality level 200 with Enhanced Validation using independent antibody verification (Demonstrating antibody specificity through the use of multiple antibodies against target in IHC or ICC.)<br>2. TOM20-rabbit (sc-11415, Santa Cruz Biotechnology)<br>(a) Website: https://www.citeab.com/antibodies/832738-sc-11415-tom20-antibody-fl-145 (antibody discontinued)<br>(b) 756 citations for this product; eg reference: Klevanski, M. et al. Automated highly multiplexed super-resolution imaging of protein nano-architecture in cells and tissues. Nat. Commun. 11, 1552 (2020).<br>3. Bassoon-mouse (SAP7F407, Enzo Life Sciences)<br>(a) Vendor website: https://www.enzolifesciences.com/ADI-VAM-PS003/bassoon-monoclonal-antibody-sap7f407/

(b) Validated in electron microscopy, IF, IHC, IP, WB
(c) Selected reference: Yamamoto, Y., Moriai, H., Yokoyama, T. & Nakamuta, N. Immunohistochemical distribution of proteins involved in glutamate release in subepithelial sensory nerve endings of rat epiglottis. Histochem. Cell Biol. 157, 51–63 (2022).
4. Homer1-rabbit (160003, Synaptic Systems)
(a) Vendor website: https://sysy.com/product/160003
(b) Validated in WB, IP, ICC, IHC, IHC-P, ELISA
(c) Selected reference: Prieto, M. et al. Missense mutation of Fmr1 results in impaired AMPAR-mediated plasticity and socio-cognitive deficits in mice. Nat. Commun. 12, 1557 (2021).
5. Glial Fibrillary Acidic Protein-chicken (173006; Synaptic Systems)
(a) Vendor website: https://www.sysy.com/product/173006
(b) Validated in ICC, IHC, IHC-P
(c) Lyu, Z. et al. A neurovascular-unit-on-a-chip for the evaluation of the restorative potential of stem cell therapies for ischaemic stroke. Nat Biomed Eng 5, 847–863 (2021).
6. Neurofilament M-mouse (171241, Synaptic Systems)
(a) Vendor website: https://www.sysy.com/product/171241
(b) Validated in WB, ICC, IHC, IHC-P
(c) Barry, D. M., Millecamps, S., Julien, J.-P. & Garcia, M. L. New movements in neurofilament transport, turnover and disease. Exp. Cell Res. 313, 2110–2120 (2007).

Animals and other organisms

Policy information about studies involving animals; ARRIVE guidelines recommended for reporting animal research

Laboratory animals	Animals were kept under environmentally controlled conditions in the absence of pathogens and ad libitum access to food and water. Preparation of brain sections containing the MNTB was performed according to an established protocol (Klevanski et al., 2020) with slight modifications. Briefly, either male or female Sprague-Dawley rats (Charles River) at postnatal day 13 were anesthetised and perfused transcardially with PBS followed by 4% PFA (Sigma-Aldrich).
Wild animals	No wild animals were used in this study.
Field-collected samples	No field-collected samples were used in this study.
Ethics oversight	All experiments that involved the use of animals were performed in compliance with the relevant laws and institutional guidelines of Baden–Württemberg, Germany (protocol G-214/20) and approved by the Regierungspraesidium Karlsruhe.

Note that full information on the approval of the study protocol must also be provided in the manuscript.
